# 嵌合抗原受体T细胞靶向LMP1抗原治疗EB病毒阳性淋巴瘤的功能研究

**DOI:** 10.3760/cma.j.issn.0253-2727.2022.03.008

**Published:** 2022-03

**Authors:** 慧珍 何, 妍妍 邢, 瑜 张, 颖茜 徐, 征 田, 海燕 邢, 克晶 唐, 青 饶, 建祥 王, 敏 王

**Affiliations:** 中国医学科学院北京协和医学院血液病医院（中国医学科学院血液学研究所），实验血液学国家重点实验室，国家血液系统疾病临床医学研究中心，天津市血液病细胞治疗研究重点实验室，天津 300020 State Key Laboratory of Experimental Hematology, National Clinical Research Center for Blood Diseases, Institute of Hematology & Blood Diseases Hospital, Peking Union Medical University, CAMS & PUMC, Tianjin Key Laboratory of Cell Therapy for Blood Diseases, Tianjin 300020, China

**Keywords:** EB病毒, LMP1抗原, 嵌合抗原受体T细胞, Epstein-Barr virus, LMP1 antigen, Chimeric antigen receptor T cell

## Abstract

**目的:**

制备一种靶向LMP1抗原的嵌合抗原受体T细胞（CAR-T细胞），研究其对EB病毒（EBV）阳性淋巴瘤的免疫治疗作用。

**方法:**

应用分子克隆技术构建二代LMP1 CAR表达质粒，通过慢病毒包装体系包装病毒后感染人T细胞，获得LMP1 CAR-T细胞，体外实验验证LMP1 CAR-T细胞对感染EBV后的LMP1阳性淋巴瘤细胞的特异性细胞毒性作用。

**结果:**

①LMP1蛋白表达于EBV阳性的淋巴瘤细胞表面；②成功构建了二代LMP1 CAR慢病毒载体，感染人T细胞，获取LMP1 CAR-T细胞，感染效率大于80％；③LMP1 CAR-T细胞可特异性杀伤LMP1阳性淋巴瘤细胞，当效靶比按4∶1共培养48 h后，LMP1 CAR-T细胞对Raji细胞的杀伤作用增强，对Ramos细胞无明显杀伤作用；④与LMP1阳性淋巴瘤细胞按1∶1共培养5 h后，LMP1 CAR-T细胞处理组CD107a^+^ T细胞比例显著高于Vector-T细胞组［（13.25±2.94）％对（1.55±0.05）％，*t*＝3.972，*P*＝0.017］，脱颗粒效果增强；⑤与LMP1阳性淋巴瘤细胞共培养后，LMP1 CAR-T细胞组CD69^+^、CD25^+^ T细胞比例显高于Vector-T细胞组［（7.40±0.41）％对（3.48±0.47）％，*t*＝6.268，*P*＝0.003；（73.00±4.73）％对（57.67±2.60）％，*t*＝2.842，*P*＝0.047］，活化效应增强。⑥与LMP1阳性淋巴瘤细胞共培养后，LMP1 CAR-T细胞组细胞因子分泌增强，高于Vector-T细胞组［IFN-γ：（703±73）ng/L对（422±87）ng/L，*t*＝2.478，*P*＝0.068；TNF-α：（215±35）ng/L对（125±2）ng/L，*t*＝2.536，*P*＝0.064］。

**结论:**

该研究证实EBV阳性淋巴瘤细胞表面可特异表达LMP1蛋白，成功构建了LMP1 CAR慢病毒载体并感染人T细胞，成功获得LMP1 CAR-T细胞。体外实验证实：与LMP1阳性淋巴瘤细胞共培养后，LMP1 CAR-T细胞脱颗粒效果增强，活化效应增强，高效分泌细胞因子，可特异杀伤LMP1阳性淋巴瘤细胞，具有潜在的临床应用前景。

## Abstract (en)

**Objective:**

This study aimed to create a type of CAR-T cells that targets LMP1 antigen and study its immunotherapeutic effect on LMP1-positive hematological malignancies.

**Methods:**

To generate LMP1 CAR-T cells, a plasmid expressing LMP1 CAR was created using molecular cloning technology, and T cells were infected with LMP1 CAR lentivirus. The effects of LMP1 CAR-T cells on specific cytotoxicity against LMP1-positive tumor cell lines infected with the EB virus had been confirmed.

**Results:**

① LMP1 protein expressing on EB virus-positive lymphoma cells surface was verified. ② The LMP1 CAR-expressing plasmid was created, and LMP1 CAR-T cells were obtained by infecting T cells with a lentivirus packaging system, with an infection efficiency of more than 80％. ③LMP1 CAR-T cells have a 4∶1 effect-to-target ratio in killing LMP1-positive lymphoma cells. The killing effect of LMP1 CAR-T cells on Raji cells was enhanced after 48 h of coculture, but there was no significant killing effect on Ramos, which are LMP1-negative lymphoma cells. ④After coculture with LMP1-positive lymphoma cells at a ratio of 1∶1 for 5 h, the degranulation effect was enhanced. The proportion of CD107a^+^ T cells in the LMP1 CAR-T cell treatment group was significantly higher than that in the vector-T cell group［（13.25±2.94）％ *vs*（1.55±0.05）％, *t*＝3.972, *P*＝0.017］. ⑤After coculture with LMP1-positive lymphoma cells, the proportion of CD69^+^ and CD25^+^ T cells in the LMP1 CAR-T cell group was significantly higher than that in vector-T cell group［（7.40±0.41）％ *vs*（3.48±0.47）％, *t*＝6.268, *P*＝0.003;（73.00±4.73）％ *vs*（57.67±2.60）％, *t*＝2.842, *P*＝0.047］. ⑥After coculture with LMP1-positive lymphoma cells, cytokine secretion in the LMP1 CAR-T cell group was higher than that in the vector-T cell group［interferon-gamma:（703±73）ng/L *vs*（422±87）ng/L, *t*＝2.478, *P*＝0.068; tumor necrosis factor-alpha:（215±35）ng/L *vs*（125±2）ng/L, *t*＝2.536, *P*＝0.064］.

**Conclusion:**

In this study, we found that the LMP1 protein is only found on the surface of the EBV-positive tumor cell. Simultaneously, we created an LMP1 CAR-expressing plasmid and obtained LMP1 CAR-T cells by infecting T cells with a lentivirus packaging system. Furthermore, we demonstrated that LMP1 CAR-T cells could specifically kill LMP1-positive tumor cells in vitro. The degranulation and activation effects of LMP1 CAR-T cells were enhanced after coculture with LMP1-positive tumor cells, indicating a potential clinical application.

EB病毒（EBV）是一种双链DNA病毒，属于人类疱疹病毒，人群感染率高达90％[1]，是WHO发布的Ⅰ级致癌物[2]。该病毒感染人体后，可引发EBV相关的恶性疾病[3]。LMP1蛋白是一种六次跨膜的Ⅱ型跨膜蛋白，有386个氨基酸分子[4]，是一种EBV慢性潜伏感染时特异性表达的膜抗原，在移植后淋巴细胞增殖性疾病、霍奇金淋巴瘤、非霍奇金淋巴瘤、T/NK T细胞淋巴瘤、Burkitt淋巴瘤、胃腺癌及鼻咽癌中均可表达[5]–[6]。

迄今为止，针对EBV感染相关的疾病治疗方法众多，包括抗病毒药物，免疫佐剂，回输EBV特异性细胞毒性T细胞等[7]。但这些治疗手段疗效十分有限，仍需探索新的治疗方法。嵌合抗原受体T细胞（CAR-T细胞）免疫疗法是近年来一项新兴的免疫疗法[8]。该治疗方法通过基因重组的方式，将识别肿瘤相关抗原的单链抗体、铰链与跨膜区、胞内信号域与共刺激分子进行重组，通过慢病毒转染体系包装，感染患者自身的T细胞，获得表达抗肿瘤抗原的单链抗体的T细胞，经过大规模扩增后再回输至患者体内，可以实现靶向消除肿瘤的目的[9]。

本研究中，针对EBV阳性淋巴瘤靶向特异表达的LMP1抗原，我们制备了LMP1 CAR-T细胞进行研究，通过体外试验，初步验证了LMP1 CAR-T细胞抗EBV阳性淋巴瘤的作用。

## 材料与方法

一、主要材料

1. 细胞培养：Burkitt淋巴瘤细胞系Raji细胞为本实验室保存，人B淋巴细胞瘤细胞系Ramos细胞购买于国家生物医学实验细胞资源库，均培养于含10％胎牛血清的RPMI 1640培养基中。健康成人外周血标本取自天津市血液中心，人T细胞通过RossetteSep T细胞富集试剂、Ficoll淋巴细胞分离液富集纯化后，培养于含5％胎牛血清和50 IU IL-2的KBM581培养基中。以上细胞都培养在37 °C、5％CO_2_环境中。

2. 主要试剂：蛋白电泳凝胶试剂盒购于中国达科为生物工程有限公司；4％多聚甲醛购于中国碧云天公司；人IFN-γ、TNF-α ELISA试剂盒购于德国Novus公司；APC抗人Fab抗体、PE抗人 CD107a抗体、APC抗人CD3抗体、PE 抗人CD19抗体、PE抗人CD69抗体、PE抗人CD25抗体购于美国Biolegend公司；PE 鼠抗人IgG Fc抗体购于美国Jackson Immuno公司；抗人LMP1［H3］IgG1购于英国Absolute Antibody公司；RossetteSep T细胞富集试剂购于美国Stem Cell公司；人T细胞激活因子CD3/CD28磁珠购于美国Gibco 公司；细胞因子IL-2购自美国R&D公司；DMEM、RPMI 1640培养基购于美国Gibco公司；KBM581培养基购于美国Corning公司。

二、方法

1. Western blot验证LMP1抗原表达：使用蛋白电泳凝胶试剂盒配胶，取Raji和Ramos细胞各1×10^6^于1.5 ml EP管中，裂解煮沸后上样，经电泳、湿转、封闭后，再孵育一抗、二抗，最后化学显影。

2. 流式细胞术标记LMP1抗原：使用4％多聚甲醛常温固定10 min，再用抗人LMP1 IgG1抗体室温孵育40 min，之后使用PE标记的抗人IgG Fc 4 °C孵育30 min，用流式细胞仪检测LMP1表达。

3. 构建LMP1 CAR表达载体：通过中国专利库，查询得到靶向LMP1抗原的人源scFv核苷酸序列（专利号：CN 109400712 A），按照先可变区轻链后重链顺序排列，进一步优化CAR结构中anti-LMP1 scFv结构，通过分子克隆技术构建二代LMP1 CAR载体。

4. 慢病毒包装：LMP1 CAR质粒、Vector（pCDH-GFP）质粒与慢病毒包装质粒PMD2.0G、JA3、JA4共同转染HEK-293T细胞，待48 h后收集病毒上清，将病毒超速离心浓缩后，分装冻存−80 °C冰箱备用。

5. LMP1 CAR载体感染T细胞：浓缩后的病毒感染经CD3/CD28磁珠刺激活化的T细胞，4 d后使用抗人IgG F（ab′）_2_抗体标记T细胞表面scFv，应用流式细胞仪检测CAR的表达效率。

6. CAR-T 细胞与靶细胞共培养后检测残留的肿瘤细胞：LMP1 CAR-T、Vector-T细胞按照效靶比4∶1和8∶1分别与LMP1阳性的Raji细胞或LMP1阴性的Ramos细胞37 °C、5％ CO_2_共培养24、48 h，由于Raji与Ramos细胞稳定表达CD19抗原，且CD19抗原阳性率都高于99％，使用流式细胞术检测靶细胞中CD19阳性残余细胞比例作为肿瘤细胞残余比例。

7. 脱颗粒效果检测：LMP1 CAR-T、Vector-T细胞按照效靶比1∶1分别与LMP1阳性的Raji细胞或LMP1阴性的Ramos细胞37 °C、5％ CO_2_共培养5 h，流式细胞术检测T细胞中CD107a^+^ T细胞比例，通过CD107a^+^/CD3^+^比例反映T细胞激活率。

8. T细胞活化能力检测：LMP1 CAR-T、Vector-T细胞按照效靶比1∶1分别与LMP1阳性的Raji细胞或LMP1阴性的Ramos细胞37 °C、5％ CO_2_共培养8、48 h，流式细胞术检测T细胞中CD69阳性或CD25阳性细胞比例，反映T细胞活化率。

9. 细胞因子释放检测：LMP1 CAR-T、Vector-T细胞按照效靶比4∶1分别与LMP1阳性的Raji细胞或LMP1阴性的Ramos细胞共培养，培养条件为37 °C，5％ CO_2_，共培养48 h后，收集上清，使用ELISA试剂盒检测上清内IFN-γ、TNF-α表达情况。

10. 统计学处理：采用 GraphPad Prism 8软件进行统计学分析。结果以均数±标准差表示，组间比较采用独立样本*t*检验分析。

## 结果

1. EBV阳性淋巴瘤细胞特异表达LMP1蛋白：LMP1抗原结构见图1A，Western blot及流式细胞术结果证实EBV阳性Raji细胞表达LMP1蛋白，而EBV阴性Ramos细胞不表达LMP1蛋白（图1B、1C）。

**图1:**
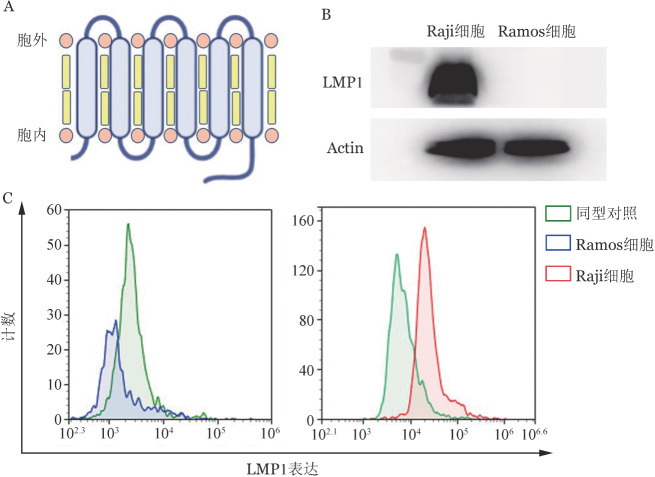
LMP1蛋白示意图及表达检测 A：LMP1蛋白结构示意图；B：Western blot检测LMP1蛋白表达；C：流式细胞术检测LMP1蛋白表达

2. LMP1 CAR质粒构建及T细胞感染：LMP1 CAR载体示意图见图2A。分选的健康人外周血CD3^+^ T细胞用抗人CD3/CD28磁珠刺激培养，24 h后感染慢病毒体系包装的二代LMP1 CAR表达载体，从而获得LMP1 CAR-T细胞，通过使用IgG Fab抗体标记LMP1 CAR-T细胞表面表达的抗LMP1抗原的scFv，测得LMP1 CAR表达载体的感染效率可达到80％。培养12 d后LMP1 CAR-T细胞可扩增至100倍（图2B）。

**图2:**
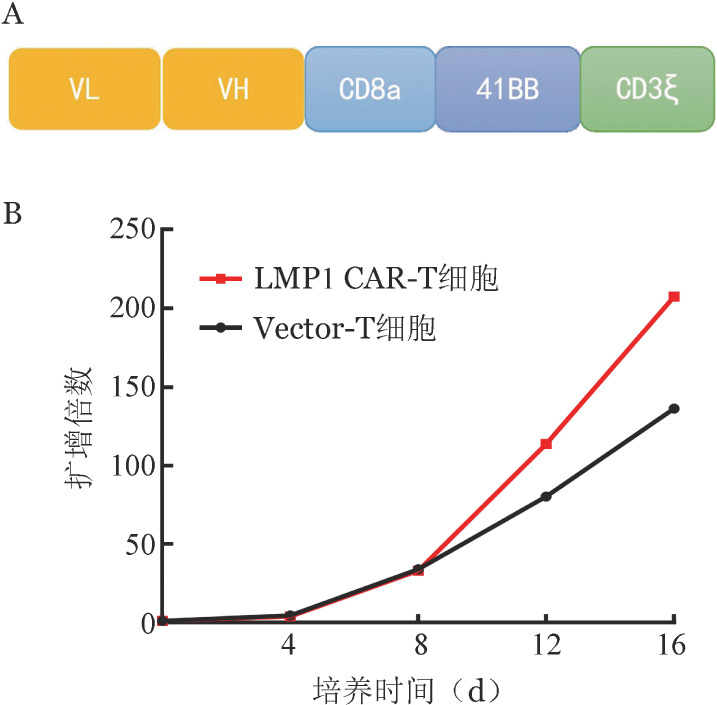
LMP1嵌合抗原受体T细胞（CAR-T细胞）的制备与扩增 A：LMP1 CAR结构示意图；B：LMP1 CAR-T细胞体外扩增曲线

3. LMP1 CAR-T细胞对靶细胞的特异杀伤作用：LMP1 CAR-T、Vector-T细胞分别与Raji细胞或Ramos细胞以效靶比4∶1和8∶1共培养24、48 h，流式细胞术检测共培养体系中残余靶细胞比例，以此反映LMP1 CAR-T细胞的杀伤能力。结果显示，与Raji细胞共培养24 h，Vector-T细胞对Raji细胞无明显杀伤作用，而LMP1 CAR-T细胞具有明显的杀伤作用。与Raji细胞共培养48 h，与Vector-T细胞相比，LMP1 CAR-T细胞杀伤作用更加显著。而与Ramos细胞共培养时，LMP1 CAR-T细胞与Vector-T细胞不能有效杀伤肿瘤细胞（图3）。结果表明，LMP1 CAR-T细胞对Raji细胞具有特异性杀伤作用，而对Ramos细胞无杀伤作用。

**图3:**
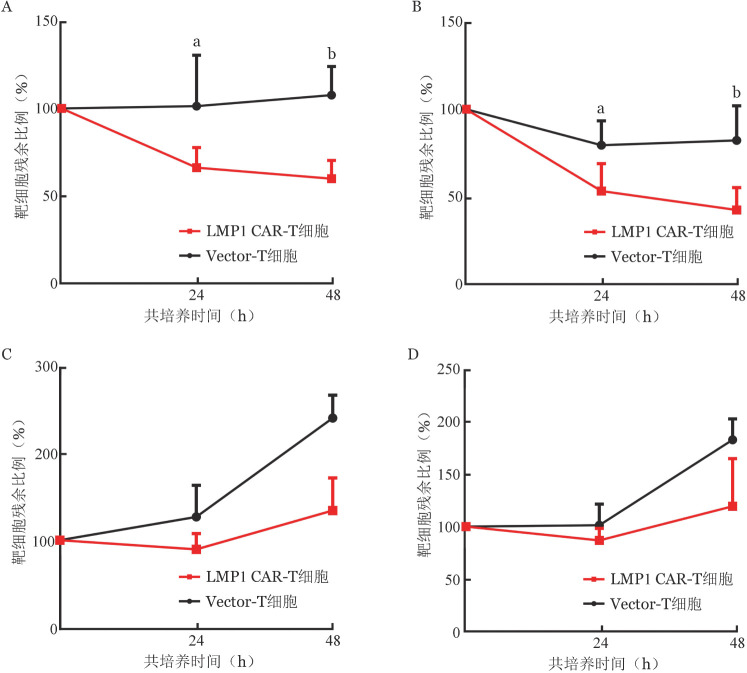
LMP1嵌合抗原受体T细胞（CAR-T细胞）特异性杀伤肿瘤细胞作用（实验重复3次，^a^*P*<0.05，^b^*P*<0.01） A：效靶比4∶1与Raji细胞共培养；B：效靶比8∶1与Raji细胞共培养；C：效靶比4∶1与Ramos细胞共培养；D：效靶比8∶1与Ramos细胞共培养

4. LMP1 CAR-T细胞脱颗粒效果：将LMP1 CAR-T细胞与Raji或Ramos细胞按效靶比1∶1共培养5 h，流式细胞术检测CD107a^+^ T细胞在T细胞中的比例。结果显示，在Raji细胞中，LMP1 CAR-T细胞处理组CD107a^+^ T细胞比例显著高于Vector-T细胞组［（13.25±2.94）％对（1.55±0.05）％，*t*＝3.972，*P*＝0.017］；而在Ramos细胞中，LMP1 CAR-T细胞组CD107a^+^ T细胞比例高于Vector-T细胞组［（9.44±1.92）％对（0.97±0.18）％，*t*＝4.401，*P*＝0.046］，但仍低于LMP1 CAR-T细胞与Raji细胞共培养组。提示与特异性抗原的靶细胞共培养5 h后，LMP1 CAR-T细胞显著提高T细胞激活率，脱颗粒效果增强，有利于更好地发挥抗肿瘤的效果。

5. 活化功能检测：将LMP1 CAR-T细胞、Vector-T细胞分别与LMP1阳性Raji细胞、LMP1阴性Ramos细胞按效靶比1∶1共培养8 h，检测早期活化标志CD69^+^ T细胞比例；共培养48 h，检测晚期活化标志CD25^+^ T细胞比例。结果显示，LMP1 CAR-T细胞与Raji细胞共培养后，CD69^+^、CD25^+^ T细胞比例显著高于Vector-T细胞组［（7.40±0.41）％对（3.48±0.47）％，*t*＝6.268，*P*＝0.003；（73.00±4.73）％对（57.67±2.60）％，*t*＝2.842，*P*＝0.047］。与Ramos细胞共培养后，LMP1 CAR-T细胞组与Vector-T 细胞组CD69^+^、CD25^+^ T细胞比例差异无统计学意义［（1.99±0.43）％对（1.89±0.48）％，*t*＝0.155，*P*＝0.884；（58.00±3.51）％对（50.00±1.00）％，*t*＝2.191，*P*＝0.094］。提示，与特异性抗原的靶细胞共培养后，活化的LMP1 CAR-T细胞比例增高。

6. 细胞因子释放检测：将LMP1 CAR-T细胞、Vector-T细胞分别与LMP1阳性Raji细胞、LMP1阴性Ramos细胞按效靶比4∶1共培养48 h后，ELISA法检测细胞因子IFN-γ、TNF-α的表达情况。结果显示，在Raji细胞共培养体系中，LMP1 CAR-T细胞组IFN-γ、TNF-α表达水平均高于Vector-T细胞组［（703±73）ng/L对（422±87）ng/L，*t*＝2.478，*P*＝0.068；（215±35）ng/L对（125±2）ng/L，*t*＝2.536，*P*＝0.064］，在0.07的水平上有统计学意义；而在Ramos细胞共培养体系中，LMP1 CAR-T细胞组与Vector-T细胞组IFN-γ、TNF-α表达水平差异无统计学意义［（220±57）ng/L对（169±28）ng/L，*t*＝0.799，*P*＝0.469；（82±32）ng/L对（55±12）ng/L，*t*＝0.769，*P*＝0.485］。反映出与LMP1阳性淋巴瘤细胞共培养时，LMP1 CAR-T细胞抗肿瘤活性增强。

## 讨论

自EBV首次通过电镜从非洲Burkitt淋巴瘤细胞中发现至今已经60余年[10]，临床治疗上EBV阳性的淋巴瘤还是以化疗与放疗联合治疗为主，联合治疗霍奇金淋巴瘤的5年生存率为80％～90％，但该方式易导致严重长期毒性，包括继发性恶性肿瘤、心肺毒性、甲状腺功能减低、不孕症等不良反应，且对于治疗后复发的患者预后不佳[11]。近年来，免疫检查点抑制剂在临床上逐见成效，如PD-1可有效改善患者免疫抑制的情况[12]，体外扩增患者来源的EBV特异性T细胞再回输的治疗方式也同样有效，特别是针对淋巴细胞增殖性疾病[13]。然而，这些治疗方法仍然无法满足全面、有效治疗的需求。

CAR-T细胞免疫治疗是一种可以实现靶向抗肿瘤的治疗方式[14]。EBV感染人体后，感染的B细胞和扁桃体上皮细胞在慢性潜伏期均可表达EBV相关遗传物质[15]，此前已有针对鼻咽癌的LMP1 CAR-T研究[16]，此外，还有针对鼻咽癌的LMP2a CAR-T[17]、针对EBV感染的gp-350 CAR-T[18]等研究（表1）。这些CAR-T细胞都取得不错的抗肿瘤效果，但与靶向病毒颗粒蛋白gp350相比，靶向潜伏膜蛋白的CAR-T细胞治疗安全性更好。

**表1:** 靶向EB病毒（EBV）阳性疾病的嵌合抗原受体T细胞（CAR-T细胞）治疗策略

文献来源	CAR-T细胞类型	靶向的肿瘤类型	效果及安全性
Kieser等[16]	LMP1 CAR-T	靶向EBV阳性鼻咽癌	有效，无GVHD发生
陈渊等[17]	LMP2a CAR-T	靶向EBV阳性鼻咽癌	有效，无GVHD发生
Slabik等[18]	gp350 CAR-T	靶向EBV急性感染的淋巴增殖性疾病	有效，但CD4^+^ CAR-T细胞可发生异种GVHD

注：GVHD：移植物抗宿主病

基于以上研究背景，本研究的创新之处在于将EBV感染后特异性表达的LMP1抗原作为靶点，结合CAR-T靶向治疗的治疗方式，验证靶向EBV阳性的淋巴瘤的治疗作用。

综上，我们成功构建了LMP1 CAR表达载体，LMP1 CAR的表达效率达到80％以上，制备出LMP1 CAR-T细胞，并通过将LMP1 CAR-T细胞与LMP1阳性淋巴瘤细胞共培养的方式，证实了LMP1 CAR-T细胞可被特异性激活、高效分泌细胞因子，对LMP1阳性淋巴瘤具有明显抗肿瘤能力，且对LMP1抗原阴性淋巴瘤无明显特异杀伤能力，表现出一定的安全性，为未来临床治疗EBV阳性淋巴瘤提供新的治疗思路。
